# Supplementary material for: The mediation effects of COVID-19-related traumatic stress symptoms and mentalization on the relationship between perceived stress and psychological well-being in healthcare workers transitioning to a post-pandemic world
Source: PLoS One. 2024 Sep 4;19(9):e0309561. doi: 10.1371/journal.pone.0309561 (PMC11373818; doi:10.1371/journal.pone.0309561)
Supplement: S1 Appendix — (DOCX) [file pone.0309561.s001.docx]

**Appendix of measures**

**The Perceived Stress Scale (PSS)**

For each question choose from the following alternatives:0= Never, 1=Almost never, 2=Sometimes, 3=Fairly often, 4=Very often

1. In the last month, how often have you been upset because of something that happened unexpectedly?

2. In the last month, how often have you felt that you were unable to control the important things in your life?

3. In the last month, how often have you felt nervous and "stressed"?

4. In the last month, how often have you dealt successfully with irritating life hassles?

5. In the last month, how often have you felt that you were effectively coping with important changes that were occurring in your life?

6.In the last month, how often have you felt confident about your ability to handle your personal

problems?

7.In the last month, how often have you felt that things were going your way?

8.In the last month, how often have you found that you could not cope with all the things that you had to do?

9. In the last month, how often have you been able to control irritations in your life?

10. In the last month, how often have you felt that you were on top of things?

**The Impact of Event Scale-Revised (IES-R)**

For each question choose from the following alternatives:0= Not at all, 1 = A little bit, 2 = Moderately, 3 = Quite a bit, 4 = Extremely.

1. Any reminder brought back feelings about it.

2. I had trouble staying asleep.

3. Other things kept making me think about it.

4. I felt irritable and angry.

5. I avoided letting myself get upset when I thought about it or was reminded of it.

6. I thought about it when I didn’t mean to.

7. I felt as if it hadn’t happened or wasn’t real.

8. I stayed away from reminders of it.

9. Pictures about it popped into my mind.

10. I was jumpy and easily startled.

11. I tried not to think about it.

12. I was aware that I still had a lot of feelings about it, but I didn’t deal with them.

13. My feelings about it were kind of numb.

14. I found myself acting or feeling like I was back at that time.

15. I had trouble falling asleep.

16. I had waves of strong feelings about it.

17. I tried to remove it from my memory.

18. I had trouble concentrating.

19. Reminders of it caused me to have physical reactions, such as sweating, trouble breathing, nausea, or a pounding heart.

20. I had dreams about it.

21. I felt watchful and on-guard.

22. I tried not to talk about it.

**The Reflective Functioning Questionnaire (RFQ-8)**

For each question choose from the following alternatives:1 = strongly disagree, 7 = strongly agree

1.People's thoughts are a mystery to me.

2.I don't always know why I do what I do.

3.When I get angry, I say things without really knowing why I am.

4.When I get angry, I say things that I later regret.

5.If I feel insecure, I can behave in ways that put others' backs up.

6.Sometimes I do things without really knowing why.

7.People's thoughts are a mystery to me.

8.Strong feelings often cloud my thinking.

The Scale of Psychological Well-being (PWB)

For each question choose from the following alternatives:1 = strongly agree, 2 = somewhat agree, 3 = a little agree, 4 = neither agree or disagree, 5 = a little disagree, 6 = somewhat disagree, 7 = strongly disagree

1.I like most parts of my personality.

2.When I look at the story of my life, I am pleased with how things have turned out so far.

3.Some people wander aimlessly through life, but I am not one of them.

4.The demands of everyday life often get me down.

5.In many ways, I feel disappointed about my achievements in life.

6.Maintaining close relationships has been difficult and frustrating for me.

7.I live life one day at a time and don't really think about the future.

8.In general, I feel I am in charge of the situation in which I live.

9.I am good at managing the responsibilities of daily life.

10.I sometimes feel as if I've done all there is to do in life.

11.For me, life has been a continuous process of learning, changing, and growth.

12.I think it is important to have new experiences that challenge how I think about myself and the world.

13.People would describe me as a giving person, willing to share my time with others.

14.I gave up trying to make big improvements or changes in my life a long time ago.

15.I tend to be influenced by people with strong opinions.

16.I have not experienced many warm and trusting relationships with others.

17.I have confidence in my own opinions, even if they are different from the way most other people think.

18.I judge myself by what I think is important, not by the values of what others think is important.
