# Supplementary material for: The mediation effects of COVID-19-related traumatic stress symptoms and mentalization on the relationship between perceived stress and psychological well-being in healthcare workers transitioning to a post-pandemic world
Source: PLoS One. 2024 Sep 4;19(9):e0309561. doi: 10.1371/journal.pone.0309561 (PMC11373818; doi:10.1371/journal.pone.0309561)
Supplement: Attachment — Submitted filename: Responses to reviewers.docx [file pone.0309561.s003.docx]

**Responses to reviewers**

Reviewer #1: The research paper under discussion aims to explore the effects of perceived stress and COVID-19-related traumatic stress symptoms on healthcare workers' psychological wellbeing, and how mentalization might mediate this relationship. The paper defines key concepts such as perceived stress, traumatic stress symptoms, and mentalization, which aids in understanding their role in the study. It is a well written paper. My only critique would be that the paper's focus on collective trauma without considering other stressors impacting healthcare workers' psychological wellbeing. It is critical to evaluate a range of factors, such as individual coping mechanisms, social support, and workplace environment, to provide a holistic understanding of the wellbeing of healthcare workers.

*Author response: Thank you for this excellent suggestion. We have reframed and added content at the beginning of the introduction (line 62-140) before introducing collective trauma and other key concepts. Various factors were also emphasized in the discussion (line 695-698).We think the revision should be able to provide a more holistic view of understanding the psychological well-being of HCWs before focusing on collective trauma, perceived stress and mentalization. Thank you again for taking the time to review our manuscript.*

Reviewer #2: Excellent manuscript with clear explanation of context, scales and results. I would think that post traumatic symptoms and mentalization would mediate the effects on psychological well being of perceived stress but it is good to see it being demonstrated in your results.

*Author response: Thank you so much for your time to review our manuscript.*

ACADEMIC EDITOR:

The authors have submitted a good manuscript. However, as noted by reviewers some edits are recommended as notes: "My only critique would be that the paper's focus on collective trauma without considering other stressors impacting healthcare workers' psychological wellbeing. It is critical to evaluate a range of factors, such as individual coping mechanisms, social support, and workplace environment, to provide a holistic understanding of the wellbeing of healthcare workers." If these revisions are incorporated the manuscript can be considered for publication

*Author response: please see response to reviewer 1. Changes have been made in introduction and discussion of the manuscript.*

Journal requirements:

When submitting your revision, we need you to address these additional requirements.

1. Please ensure that your manuscript meets PLOS ONE's style requirements, including those for file naming.

*Author response: we have reformatted the manuscript according to the templates and style guideline provided. Tables were added and fonts were adjusted in the main text.*

2. We noticed you have some minor occurrence of overlapping text with the following previous publication(s)(but not limited to these, which needs to be addressed:

a) Lassri, D. and Desatnik, A., 2020. Losing and regaining reflective functioning in the times of COVID-19: Clinical risks and opportunities from a mentalizing approach. Psychological Trauma: Theory, Research, Practice, and Policy, 12(S1), p.S38.
b) Zhou, T., Xu, C., Wang, C. et al. Burnout and well-being of healthcare workers in the post-pandemic period of COVID-19: a perspective from the job demands-resources model. BMC Health Serv Res 22, 284 (2022).<https://doi.org/10.1186/s12913-022-07608-z>

In your revision ensure you cite all your sources (including your own works), and quote or rephrase any duplicated text outside the methods section. Further consideration is dependent on these concerns being addressed.

*Author response: both references mentioned have been fixed in the manuscript, direct quotes were added for reference (a), reference (b) has also been added. All the references were checked and reformatted according to the journal requirement.*

3. PLOS requires an ORCID iD for the corresponding author in Editorial Manager on papers submitted after December 6th, 2016. Please ensure that you have an ORCID iD and that it is validated in Editorial Manager. To do this, go to ‘Update my Information’ (in the upper left-hand corner of the main menu), and click on the Fetch/Validate link next to the ORCID field.

This will take you to the ORCID site and allow you to create a new iD or authenticate a pre-existing iD in Editorial Manager.

*Author response: ORCID has been added in the profile.*

4. Your ethics statement should only appear in the Methods section of your manuscript. If your ethics statement is written in any section besides the Methods, please delete it from any other section.

*Author response: this has been fixed.*

5. Please include a separate caption for each figure in your manuscript.

*Author response: this has been fixed in the manuscript and figures uploaded. Captions have been added, thank you for noticing the missing captions.*

6. Please include captions for your Supporting Information files at the end of your manuscript, and update any in-text citations to match accordingly.

*Author response: S1 Appendix was added to the main manuscript.*

7. Please review your reference list to ensure that it is complete and correct. If you have cited papers that have been retracted, please include the rationale for doing so in the manuscript text, or remove these references and replace them with relevant current references. Any changes to the reference list should be mentioned in the rebuttal letter that accompanies your revised manuscript. If you need to cite a retracted article, indicate the article’s retracted status in the References list and also include a citation and full reference for the retraction notice.

*Author response: All the references were checked and reformatted according to the journal requirement.*
